# Expanding the tagging toolbox for visualizing translation live

**DOI:** 10.1042/BCJ20240183

**Published:** 2025-01-31

**Authors:** Rhiannon M. Sears, Nathan L. Nowling, Jake Yarbro, Ning Zhao

**Affiliations:** 1 Department of Biochemistry and Molecular Genetics, University of Colorado-Anschutz Medical Campus, Aurora, CO, U.S.A

## Abstract

Translation is a highly regulated process that includes three steps: initiation, elongation, and termination. Tremendous efforts have been spent to study the regulation of each translation step. In the last two decades, researchers have begun to investigate translation by tracking it in its native and live intracellular environment with high spatiotemporal resolution. To achieve this goal, a handful of tagging tools have been developed that can distinguish nascent chains from previously synthesized mature proteins. In this review, we will focus on these tagging tools and describe their development, working mechanisms, and advantages and drawbacks in tracking translation in live mammalian cells and organisms. In the second part of the review, we will summarize novel discoveries in translation by a recently developed nascent polypeptide tracking technology using tandem epitope tag array tagging tools. The superior spatiotemporal resolution of this technology enables us to directly and continuously track nascent chains live and thus reveal preferred translation location and timing, as well as the kinetics of canonical and noncanonical translation, translation bursts, ribosome quality control, and nonsense-mediated mRNA decay. In the future, we expect more tagging tools to be developed that allow us to track other regulation processes of a protein, such as folding, modifications, and degradation. With the expanding tagging toolbox, there is potential that we can track a protein from translation to degradation to fully understand its regulation in a native live cell environment.

## Introduction

Protein biogenesis, the translation process in the central dogma of molecular biology, requires the synchronization of many molecules, including ribosomes, tRNAs, mRNAs, translation factors, and others. The elaborate process of translation is broken into three steps: initiation, elongation, and termination. Translation starts from the assembly of 43S preinitiation complexes and the subsequent recognition of start codons, followed by the recruitment of 60S ribosomal subunits and the assembly of 80S ribosomes [1]. Next, the assembled 80S ribosomes begin decoding mRNAs in triple nucleotides to synthesize nascent polypeptides until they encounter stop codons. The stop codons cause the dissociation of nascent polypeptides from the ribosomes through the exit tunnel [2]. Each step in translation is highly regulated to maintain proper protein homeostasis.

**Figure 1:**
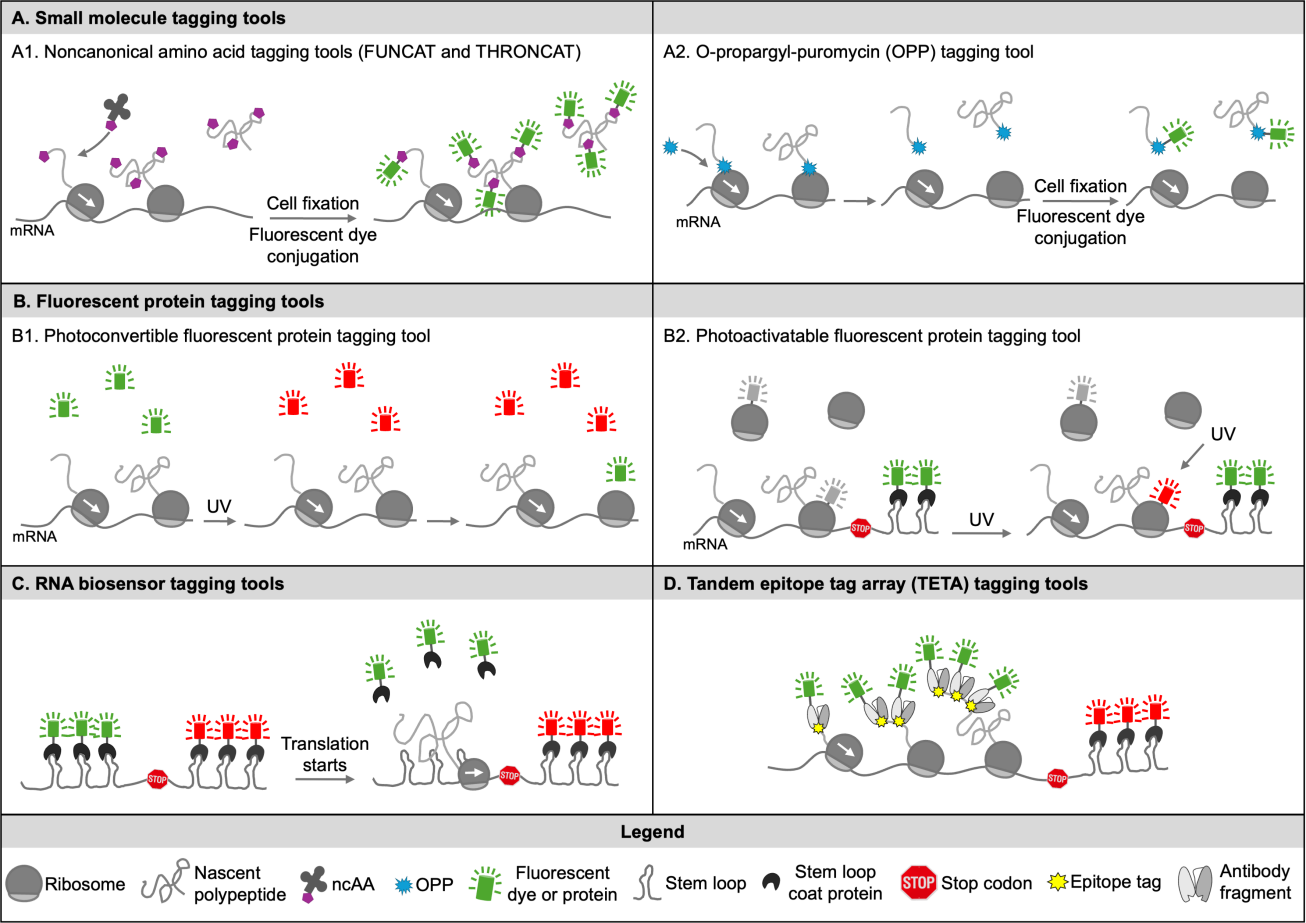
Tagging tools for visualizing translation in live cells. **(A)** Small molecule tagging tools. These tools incorporate small molecules containing either an alkyne or azide group into nascent polypeptide chains in live cells. Then, the cells are fixed, and the nascent chains are conjugated with fluorescent dyes through click chemistry between the incorporated small molecules and the fluorescent dyes. (A.1) FUNCAT and THRONCAT incorporate a methionine analog and a threonine analog into nascent chains. (A.2) O-propargyl-puromycin (OPP) is incorporated into nascent chains. **(B)** Fluorescentfluorescent protein tagging tools. (B.1) Photoconvertible fluorescent protein tagging tool. Previously synthesized photoconvertible fluorescent proteins are converted from green to red using UV light, resulting in newly synthesized proteins in green. (B.2) Photoactivatable fluorescent protein tagging tool. Ribosomes are tagged with a photoactivatable fluorescent protein, which can be photoactivated by UV light. Translating mRNAs are identified by co-localized mRNAs and ribosomes. **(C)** RNA biosensor tagging tools. These tagging tools implement two orthogonal stem-loops or RNA aptamers/stem-loops in the ORF and 3’ UTR, respectively. Non-translating mRNAs are shown as co-localized two-color puncta. When translation starts, ribosomes are loaded on the mRNAs and knock off the coat proteins bound to the stem-loops or the fluorescent dyes bound to the RNA aptamers in the ORF, resulting in one-color fluorescent puncta of translating mRNAs. **(D)** Tandem epitope tag array (TETA) tagging tools use fluorescent antibody fragments to light up nascent polypeptide chains upon binding the cognate TETA at the N-terminus of a reporter.

Translation has been investigated for several decades in great detail. However, along with the advances of novel technologies, the field continues generating new concepts and challenging the current and widely accepted paradigms. Traditional translation studies quantify final protein products using classical biochemistry assays such as western blots, luciferase assays, and fluorescent protein assays. These biochemistry assays are still widely used for studying translation. However, the main drawback of these approaches is that they cannot separate the newly synthesized proteins from the previously made proteins, which leaves the actual translation process in the dark. In the last two decades, researchers have begun investigating translation by tracking it in live cells and in real time. This technology requires tagging tools that can distinguish nascent polypeptide chains from mature proteins or active translating mRNAs from non-translating mRNAs in a single cell. In this review, we will focus on this kind of tagging tools that enable us to track translation in live cells and, therefore, to study when, where, and how mRNAs are translated into proteins with high spatiotemporal resolution. We have categorized these tools into four classes: (1) small molecule tagging tools, (2) fluorescent protein tagging tools, (3) RNA biosensor tagging tools, and (4) tandem epitope tag array (TETA) tagging tools, as illustrated in Figure 1. For each class of tagging tools, we will discuss their development and working mechanisms, as well as their pros and cons in tracking translation live.

**Figure 2:**
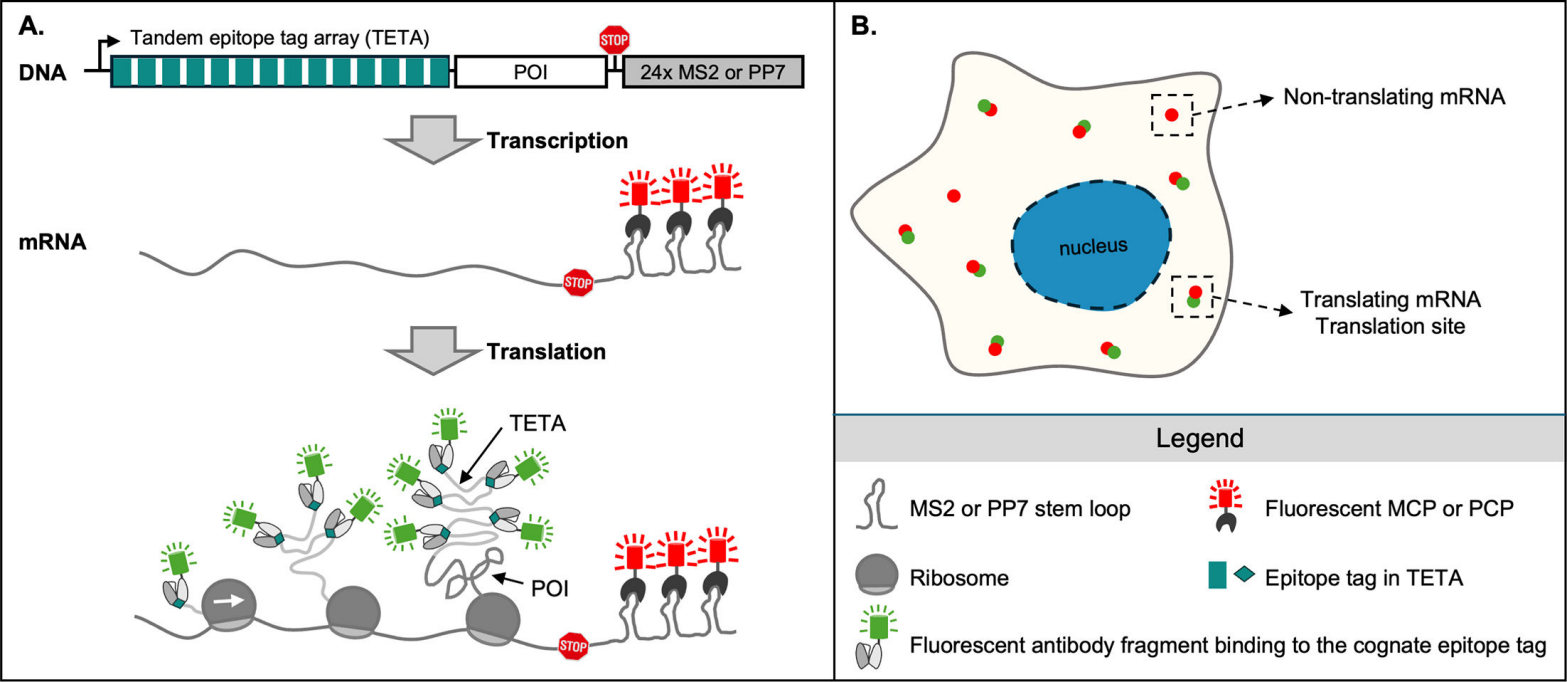
Nascent polypeptide tracking (NPT) using tandem epitope tag array (TETA) tagging tools. (**A**) The NPT technology. The NPT reporter contains a TETA at the N-terminus of the POI and 24xMS2 or PP7 RNA stem-loops in the 3‘ UTR. After transcription, the 24xMS2 or PP7 RNA stem-loops are accessible in the transcribed mRNAs. Then, the co-expressed fluorescent coat proteins bind to the stem-loops and light up mRNAs in one color (red). When translation starts, TETAs are translated first. The co-expressed fluorescent cognate antibody fragments bind the TETAs and light up nascent chains in a different color (green). (**B**) Identification of translation sites using NPT in live cells. Translation sites or translating mRNAs in cells are shown as co-localized two-color (red and green) puncta as illustrated in the lower spot. Non-translating mRNAs are shown as one-color (red) puncta.

**Figure 3:**
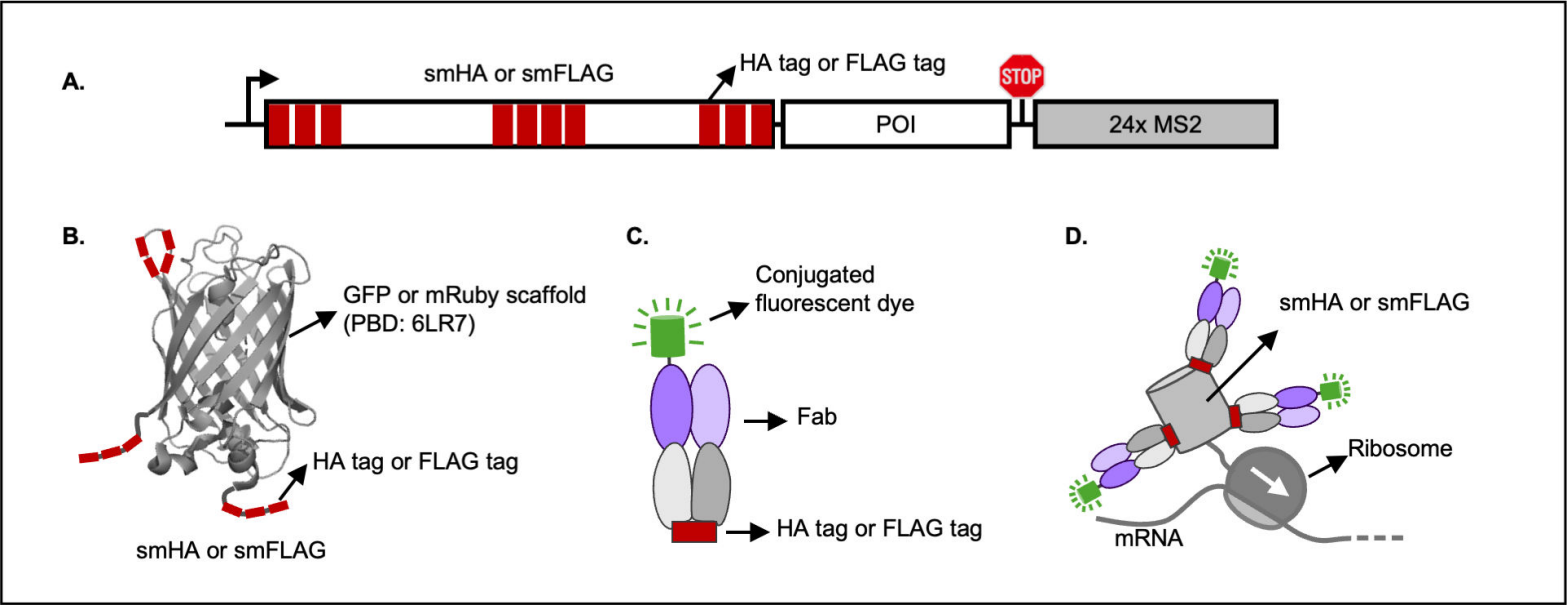
Nongenetically encodable TETA tagging tools. (**A**) The NPT reporter. The reporter contains a smHA or smFLAG (10xHA tag or FLAG tag, shown in red, embedded in a GFP or mRuby β-barrel scaffold) at the N-terminus of the POI and 24xMS2 in the 3‘ UTR. (**B**) The structure of smHA or smFLAG. The HA tags or FLAG tags are shown in red. The β-barrel structure (PDB: 2B3P6LR7) is gray. Three tags are inserted at both the N-terminus and C-terminus, and four tags are in the loop of the β-barrel structure. (**C**) A fluorescent Fab binds an HA tag or FLAG tag. (**D**) A smHA or smFLAG tagged nascent chain is lit up by fluorescent anti-HA Fabs or anti-FLAG Fabs.

**Figure 4:**
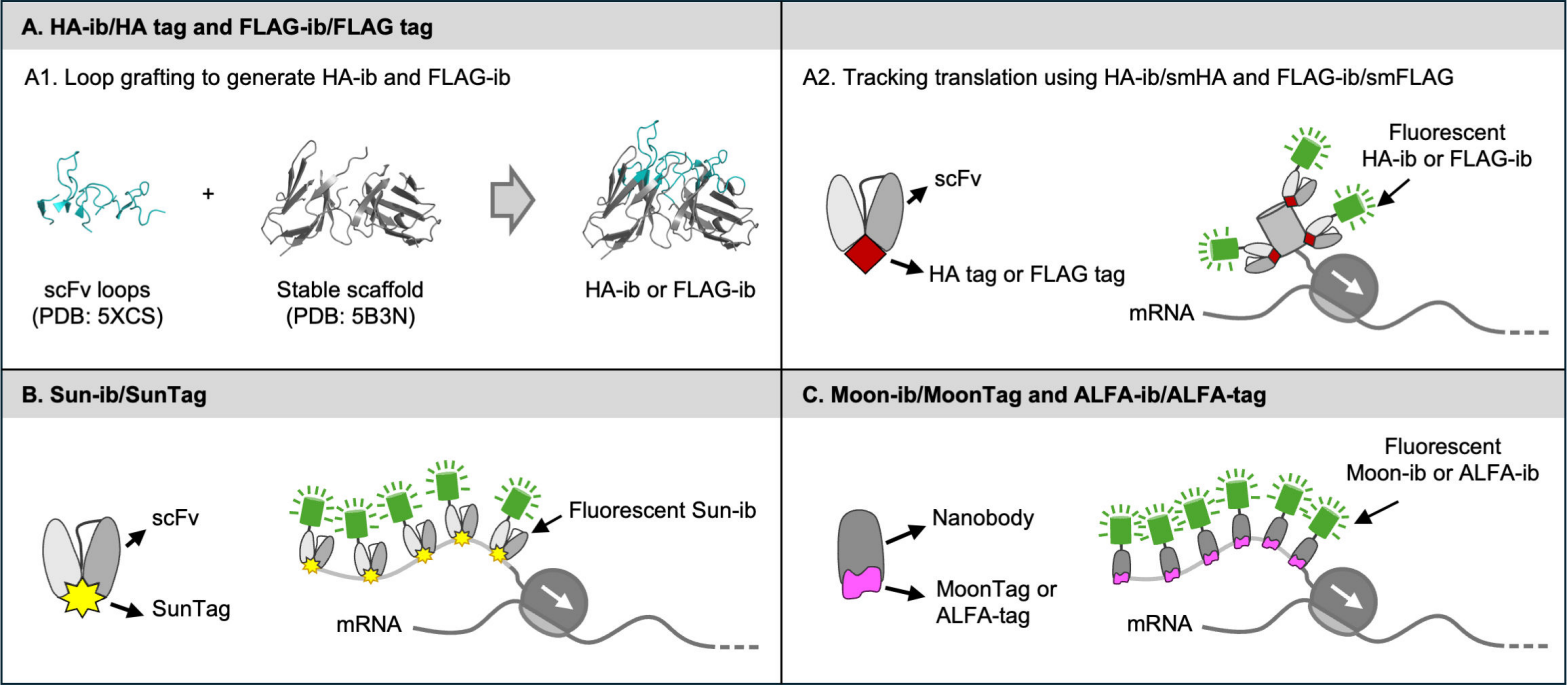
Genetically encodable TETA tagging tools. (**A**) HA-ib/HA tag and FLAG-ib/FLAG tag. (A.1) The loop grafting technique of generating the HA-ib and FLAG-ib (PDB: 5XCS and 5B3N and 5XCS). (A.2) A smHA- or smFLAG- tagged nascent chain is lit up by fluorescent HA-ibs or FLAG-ibs. (**B**) Sun-ib/SunTag. A SunTag array -tagged nascent chain is lit up by fluorescent Sun-ibs. (**C**) Moon-ib/MoonTag and ALFA-ib/ALFA-tag. A MoonTag or ALFA-tag array tagged nascent chain is lit up by fluorescent Moon-ibs or ALFA-ibs.

In the second part of the review, we will focus on the class of TETA tagging tools (Figure 1D) and a nascent polypeptide tracking (NPT) technology (Figure 2) that utilizes the TETA tagging tools. The NPT technology allows continuous and direct visualization of translation in live cells and shows superior spatiotemporal resolution compared with the technologies using other tagging tools. Therefore, the NPT technology and the TETA tagging tools have become popular in visualizing translation live in the recent decade. Up to date, several TETA tagging tools are available, including HA tag [3], FLAG tag [4], SunTag [5,6], MoonTag [7], and the newly developed ALFA-tag [8,9], as shown in Figures 3 and 4. We will specifically describe the development of these tagging tools and their applications in translation tracking. Later in the review, we will focus on discussing new discoveries in translation using the NPT technology in live mammalian cells as well as in model organisms.

## The tagging toolbox for visualizing translation live

### Small molecule tagging tools

One challenge of visualizing translation is differentiating newly synthesized proteins from previously produced ones. Pulse-chase is a traditional approach that incorporates radioactively labeled amino acids into the newly synthesized proteins in a short period (pulse) and returns the cells to a normal medium for a various length of time (chase) until resolving the nascent proteins on an SDS-PAGE gel. While translation has been explored at a temporal level using this approach, direct visualization of these nascent proteins *in situ* remained elusive. With the rise of a noncanonical amino acid (ncAA) incorporation technology, two novel small molecule tagging tools have been developed for directly visualizing nascent proteins *in situ*. One is named fluorescent noncanonical amino acid tagging tool (FUNCAT) [10,11] and the other is named threonine-derived noncanonical amino acid tagging tool (THRONCAT) [12]. Both apply the copper–catalyzed azide–alkyne cycloaddition click chemistry [13,14] to attach fluorescent dyes to the ncAA-incorporated nascent proteins (Figure 1A1).

Specifically, the FUNCAT technology incorporates noncanonical methionine analogs, such as homopropargylglycine (Hpg) containing an alkyne moiety or azidohomoalanine (Aha) containing an azide moiety, into nascent proteins *in vivo* [15,17], while the THRONCAT technology incorporates a threonine analog β-ethynylserine that contains an alkyne group. FUNCAT needs cells to be methionine depleted before adding Hpg or Aha for labeling. Methionine depletion might introduce cell stress that changes translation regulation and, thus, is less ideal for studying translation. In contrast, THRONCAT requires shorter labeling time, and more importantly, the labeling can be done in a complete medium as the incorporation efficiency of the threonine analogs is much higher than that of the methionine analogs. For FUNCAT and THRONCAT, after the ncAA incorporation, the cells must be fixed for downstream fluorescent dye attachment by click chemistry for visualization, which makes it challenging for continuous tracking of the translation process. To overcome this issue, strain-promoted azide–alkyne cycloaddition provides an alternative approach that does not require cell fixation and, therefore, enables visualizing translation in intact live cells [18,19].

Another approach to visualize nascent proteins uses the alkyne analog of puromycin O-propargyl-puromycin (OPP) [20]. Similar to puromycin, OPP forms covalent conjugates with the nascent polypeptides and inhibits translation (Figure 1A2). The incorporation of OPP into nascent polypeptides does not depend on amino acid content, and thus, no amino acid depletion is needed. However, the visualization of nascent proteins still requires cell fixation and fluorescent dye attachment by click chemistry, which hinders its application in tracking translation in real time.

These small molecule tagging tools provide an excellent method of differentiating nascent proteins from mature ones and studying global translation in diverse mammalian cells. However, they usually suffer from low spatiotemporal resolution due to either long labeling time and/or cell fixation. In addition, they cannot visualize the translation of a specific gene. To address these issues, tagging tools that can track translation with improved spatiotemporal resolution and increased specificity are needed.

### Fluorescent protein tagging tools

Another method of tracking translation uses fluorescent proteins (FPs). FPs like green fluorescent protein (GFP) and red fluorescent protein (RFP) are covalently attached to proteins of interest and, therefore, cannot differentiate nascent proteins from mature ones. Creatively, the Martin lab has applied a photoconvertible FP dendra2 [21] to study the local translation of sensorin in neurons [22]. To do this, they attached the 5′ and 3′ untranslated regions (UTRs) of sensorin to the dendra2 encoding gene to localize the mRNAs in the same spot with the endogenous sensorin mRNAs. The dendra2 can be irreversibly converted from green to red by ultraviolet (UV) illumination. They utilized this fluorescence change of the dendra2 to convert the previously synthesized dendra2 proteins to red color by UV illumination, resulting in only newly synthesized proteins remaining green (Figure 1B1). By controlling the region of UV illumination, some spatial resolution can be achieved. Since its first utilization, dendra2 has been used for the study of β-actin local translation [23], localized translation in radial glia endfeet [24], and the translation of dendritic GluN2A [25]. However, due to the nature of FP, the visualization using dendra2 requires the folding of the FP and the maturation of the fluorophore, which can take several minutes to hours. This characteristic of FPs results in missing important temporal and spatial information in these studies.

To further improve the spatiotemporal resolution of visualizing translation in live cells, the Singer lab developed a technology that co-tracks mRNAs and ribosomes [26]. This co-tracking technology enables the identification of actively translating mRNAs by the colocalization of mRNAs and ribosomes. For mRNA visualization, the Singer lab utilized an established mRNA tagging tool MS2/MCP (MS2 coat protein) [27,28]. MS2 is an RNA stem-loop derived from bacteriophage and can be recognized by its coat protein MCP. They incorporated 24 copies of MS2 stem loops into the 3′ UTR of a mRNA transcript. The reporter was co-expressed with FP-tagged MCPs to visualize single mRNAs in live cells [28,29]. For ribosome visualization, the 60S large subunit protein rpL10A was tagged with a photo-activatable RFP PATagRFP and imaged using photo-activated localization microscopy to achieve single ribosome resolution (Figure 1B2). The Singer lab used this co-tracking technology to map the translation hot spots of β-actin in mouse embryonic fibroblasts. Tracking mRNAs and ribosomes separately, they found there was a similar mobility heterogeneity in both mRNAs and ribosomes. About 56% of ribosomes and 42% of β-actin mRNAs displayed slow diffusion [26]. Treatment with puromycin or hippuristanol to interrupt mRNA–ribosome interactions increased the mobility of both mRNAs and ribosomes, indicating the mRNAs and ribosomes with slow diffusion were more likely engaged in active translation. By co-tracking mRNAs and ribosomes, they directly observed the local translation of β-actin and found that most ribosome-bound mRNAs near focal adhesions diffused slower than those away from the focal adhesions and much slower than mRNAs without ribosomes [26]. This technology allows for identifying actively translating mRNAs by co-tracking ribosomes and mRNAs with high spatiotemporal resolution. Therefore, it is great for studying local translation in live cells.

### RNA biosensor tagging tools

The Chao lab developed an RNA biosensor to differentiate translating mRNAs from non-translating mRNAs and visualize the first round of translation in live cells [30]. They applied two pairs of orthogonal RNA stem-loops and their cognate coat proteins in this technology: MS2/MCP [27,28] and PP7/PCP (PP7 coat protein) [31]. They inserted six copies of PP7 stem-loops in the open reading frame (ORF) and 24 copies of MS2 stem-loops in the 3′ UTR. The reporter was co-expressed with MCP and PCP tagged with FPs in two different colors. The reporter mRNAs in live cells were tracked by both MCP and PCP and showed as colocalized two-color puncta, as shown in Figure 1C. When translation initiates on the reporter mRNA, the ribosome knocks off the PCPs in the ORF, resulting in one-color visualization of translating mRNAs (Figure 1C). They named this technology Translating RNA Imaging by Coat Protein Knockoff (TRICK). Using TRICK, the researchers observed that 90% of the reporter mRNAs underwent their first round of translation at steady state. Furthermore, they applied TRICK to characterize the spatiotemporal regulation of the 5′ terminal oligopyrimidine (TOP) motif when cells are under stress. The Chao lab found that the 5′ TOP TRICK reporter under stress conditions resulted in untranslated reporter mRNAs in cytoplasm as well as in stress granules (SGs) and processing bodies (P-bodies). However, only mRNAs sequestered in P-bodies formed large clusters, which are much larger than a non-5′ TOP TRICK reporter under the same cell stress. Clustering of mRNAs in P-bodies occurred upon induction of cellular stress by arsenite, and removal of the stress resulted in initiating the translation of the cytoplasmic mRNAs but not those in the P-bodies. This result suggests that P-bodies may provide a unique translation regulation of mRNAs.

Later, the Chao lab incorporated the TRICK biosensor on the mRNAs of a secreted protein, *Gaussia* luciferase, for the study of ER-localized translation [32]. Dual-labeled untranslated mRNA transcripts were detected on ER, indicating *Gaussia* luciferase mRNAs are able to be recruited to ER during the first round of translation prior to the ribosomes displacing the fluorescent signals of the PCPs in the ORF, which is consistent with the signal recognition particle (SRP)-dependent mechanism. Interestingly, a *Renilla* luciferase TRICK reporter that encodes a cytosolic protein displayed a small population of untranslated mRNAs associated with the ER, indicating the *Renilla* luciferase mRNAs can also be recruited to ER during the first round of translation before the fluorescent signal of the PCPs in the ORF is knocked off by ribosomes [32]. The *Renilla* luciferase does not contain a canonical SRP signal sequence. Therefore, the localization to ER could be done through short hydrophobic stretches in the *Renilla* luciferase, which resembles the SRP signal sequence. Then, they found that the *Gaussia* luciferase mRNAs can remain on the ER longer than the *Renilla* luciferase mRNAs, which allows for more than one round of translation [32].

Another biosensor, named nanozipper, is recently developed by the Liu lab, which allows the distinction between translating and nontranslating mRNAs [33]. The nanozipper is designed based on the RNA aptamer Broccoli that becomes intensely fluorescent when bound to a cell-permeable, fluorogenic small-molecule dye 3,5-difluoro-4-hydroxybenzylidene imidazoline (DFHBI) [34]. The nanozipper contains two complementary fragments, each of which encodes a half fragment of three split Broccoli aptamers. When co-expressed with the mRNA reporters containing nanozipper recognition sites, the two complementary RNA fragments can reassemble into three DFHBI binding sites that can be lit up by DFHBI. The researchers used five nanozippers to label a single mRNA molecule, which provided good optical stability and signal-to-noise ratio for single-molecule imaging [33]. Similar to the TRICK technology, the researchers inserted five nanozippers in the ORF and 24xPP7 stem-loops in the 3′ UTR of their translation reporter. When translation starts, the fluorescent intensity of the nanozippers decreases as the ribosome sequentially disrupts the DFHBI binding site along the translation. Using the fluorescent intensity decay of the nanozippers, they measured the first round of viral mRNA translation elongation rate as 3.1 ± 1.1 codons/sec [33].

With the development of RNA biosensor tagging tools, it was possible to indirectly visualize the translation of an individual mRNA transcript. Though these tools can only visualize the first round of translation, their superior spatiotemporal resolution has been important in understanding local translation regulation, translation kinetics, and heterogeneity in translation events.

### Tandem epitope tag array tagging tools

Tremendous efforts have been spent in directly tracking the translation of a single mRNA in live cells. A breakthrough technology was developed in 2016 when five groups independently developed a novel NPT technology by tagging the N-terminus of nascent polypeptide chains with multiple copies of small epitope tags (Figure 2A) [6,35,38]. When translation starts, the repeated epitope tags are translated first and emerge from the ribosomes, as shown in Figure 2A. Their cognate fluorescent probes in the cytoplasm can light up the nascent chains upon binding the tandem tags in a fast manner (Figure 2A). We named these repeated epitope tags along with their cognate fluorescent probes tandem epitope tag array (TETA) tagging tools (Figure 1D). Meanwhile, the translation reporters contain 24xMS2 or PP7 stem-loops in the 3′ UTR. Individual mRNAs are lit up upon fluorescent MCP/PCP binding to the MS2/PP7 repeats (Figure 2A). Translation sites can be identified as colocalized nascent polypeptide spots and mRNA spots (Figure 2B). As the fluorescent background in the cytoplasm is elevated by the freely diffused fluorescent probes, the TETAs that can recruit multiple fluorescent probes to each nascent chain are critical to increasing the signal-to-noise ratio at the translation sites. The NPT technology enables us, for the first time, to directly track translation at a single mRNA level in live cells using either confocal microscopy, total internal reflection fluorescence microscopy, or highly inclined and laminated optical sheet microscopy [39].

The NPT technology is only possible with the development of TETA tagging tools. Since 2016, the Tanenbaum lab, the Stasevich lab, the Bertrand lab, and the Dufourt lab have been developing new TETA tagging tools to track translation in multiple colors (Figures 3 and 4). Below, we will introduce the development of each tagging tool and its application in tracking translation live.

#### Nongenetically encodable fluorescent probes

**Figure 5:**
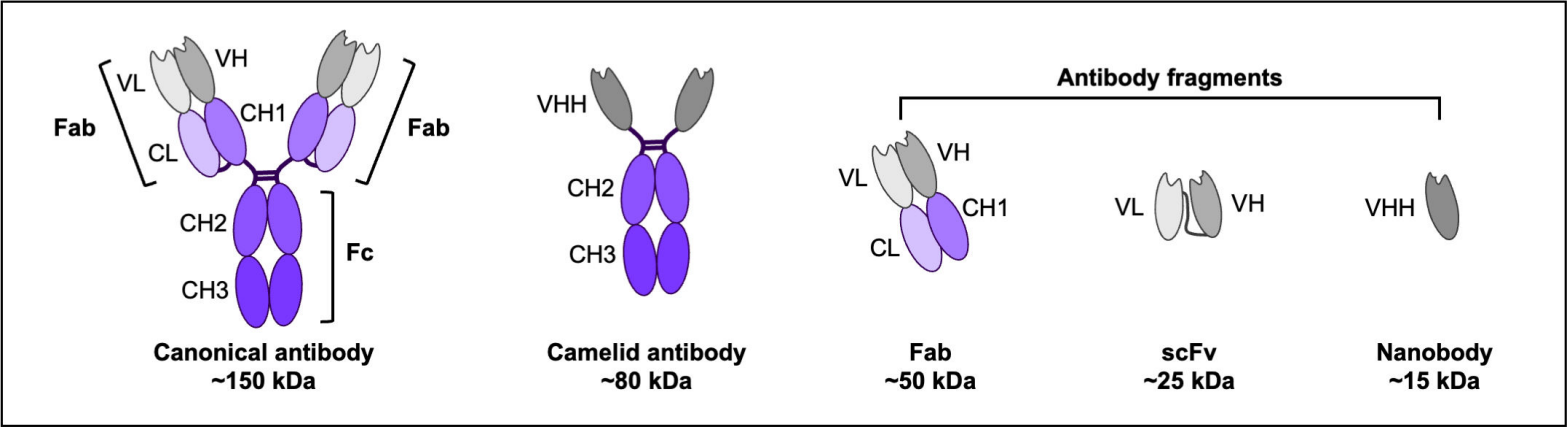
Antibodies and antibody fragments. The structures of a canonical IgG antibody and a special camelid antibody, as well as three antibody fragments: Fabs, scFvs, and nanobodies.

In 2016, the Stasevich lab tagged nascent polypeptides with short and time-tested linear epitope tags: the HA tag and the FLAG tag. As these tags are short and intrinsically disordered, they can be embedded into stable β-barrel FP scaffolds to increase their expression efficiency and stability. The Looger lab successfully incorporated 10xHA tags or 10xFLAG tags into either GFP or mRuby β-barrel scaffold and developed spaghetti monster HA (smHA) tag and spaghetti monster FLAG (smFLAG) tag (Figure 3B), which can recruit up to 10 fluorescent antibody fragments to a single protein molecule and therefore significantly enhance signal-to-noise ratio for single particle tracking [40]. The Stasevich lab then fused the smHA or smFLAG tag to the N-terminus of a POI to track nascent polypeptides in live cells (Figure 3A). As the FLAG tag and the HA tag have been used for decades in biomedical research, their cognate antibodies have been widely applied *in vitro* assays, such as ELISA, western blot, immunofluorescence, and immunoprecipitation. Although the excellent binding affinity and specificity of the antibodies have been validated for decades *in vitro*, these tags have not found their applications in live-cell imaging due to the difficulty of loading the full-length antibodies into live cells. The Stasevich lab adapted the Fab-based live endogenous modification labeling [41,46] technique to bead load [47,48] fluorescent Fabs (antigen-binding fragments, Figures 3C and 5) into live cells to visualize translation [36], which is named Fab-Bead Loading (FabBL) technique in this review. Here, we briefly describe how to visualize translation using FabBL [48]. First, the researchers generated Fabs from the commercially available purified monoclonal antibodies against the FLAG tag and the HA tag. Each antibody molecule can be digested into two identical Fabs and one Fc fragment by pepsin (Figure 5). The Fc fragments and undigested antibodies can be removed using a protein A column, as the Fc fragments bind protein A tightly and specifically. The flow-through portion from the column contains Fabs with high purity. Then, the researchers conjugated the Fabs with fluorescent dyes to generate fluorescent Fabs (Figure 3C). The fluorescent Fabs can be stored at 4°C for months. Before imaging, they use 100 µm glass beads to load the fluorescent Fabs along with translation reporter plasmids and other imaging probes into live U-2 OS cells seeded in imaging chambers. After bead loading, the glass beads are washed away, and the cells are ready for imaging. Using this unique FabBL technique, the Stasevich lab visualized translation in live cells with single mRNA resolution (Figure 3D) [36]. Moreover, they are the first to visualize translation in multiple colors by tagging distinct nascent polypeptide chains with orthogonal tagging tools: the anti-HA Fab/smHA tag and the anti-FLAG Fab/smFLAG tag [36]. The FabBL technique is later applied to study HIV frameshifting [49], internal ribosome entry site (IRES)-initiated translation [50], and translation in SGs [51] and P-bodies [52].

The Fabs delivery method, bead loading [47,48], is a cost-effective and time-efficient way to load proteins and plasmids to robust and adherent cells, such as U-2 OS cells and HeLa cells. As fluorescent Fabs are bright functional proteins, bead loading simply loads them into live cells without altering their functionality. Therefore, the cells loaded with fluorescent Fabs are ready for imaging right away. Morisaki *et al*. mentioned that the translation sites can be visualized in less than 3 seconds after injecting the fluorescent Fabs in cells [36]. Early rounds of translation can be visualized beautifully using this technique. Furthermore, the amount of loaded Fabs in each cell is fixed. Therefore, the fluorescent background in bead-loaded cells is usually lower compared with genetically encodable probes. However, the FabBL technique is harsh on cells as the glass beads physically punch holes in cells to allow the Fabs to diffuse through the plasma membrane barrier. Cancer cells like U-2 OS and HeLa can recover from the bead loading quickly. However, fragile cells, such as neurons and stem cells, cannot tolerate the force and usually die after bead loading. In addition, bead loading cannot be applied to live organisms. To overcome these limitations, a more advanced Fabs delivery system is needed. With an advanced delivery system, it will become possible to directly utilize developed and validated full-length antibodies for tracking nascent and mature proteins in live cells. Another way to overcome this Fabs delivery barrier is converting the Fabs to genetically encodable fluorescent probes, which can be easily delivered into live cells and even organisms. In the following section, we will discuss a loop grafting technique that we have applied to convert Fabs into genetically encodable intracellular antibodies (intrabodies) against the HA tag and the FLAG tag.

#### Genetically encodable fluorescent probes

The burst of developing genetically encodable fluorescent probes enables the development of NPT. As in NPT, the nascent polypeptide chains need to be tagged with TETAs to improve signal-to-noise ratio. Therefore, the cognate genetically encodable fluorescent probes that specifically and tightly bind the short epitope tags in TETAs are needed. Researchers have focused on screening and developing this type of probes, like single-chain antibody fragments (scFvs, Figure 5) and single-chain single-domain nanobodies (nbs, Figure 5). Both scFvs and nbs have the potential to retain the tight binding affinity and high specificity of the parental antibodies, assuming they fold correctly. Meanwhile, unlike secreted multi-chain antibody molecules, scFvs and nbs are single-chain molecules that can be expressed and stay in cytosol. An FP tag can be easily fused to scFvs and nbs to make them glow in live cells. These characteristics of scFvs and nbs make them great candidates for developing genetically encodable fluorescent probes to visualize translation live. These genetically encodable and functional scFvs and nbs in live cells belong to the class of intrabodies. To simplify the naming system in this review, we refer to this group of scFvs and nbs xxx-intrabodies (xxx-ib, xxx represents the name of the tag) in this review.

Many scFvs and nbs have been generated in the last 20 years. Most of them were developed through display technologies, such as yeast display and phage display. The selected hits bind to their epitope tags tightly and specifically *in vitro*. However, only few of them are functional when expressed in live cells, which is due to their folding defects in live cells. This folding defect abolishes their binding capability. A widely accepted hypothesis is the disulfide bonds in these scFvs and nbs cannot be formed in reducing cytosolic environments. Tremendous efforts have been made to rescue their binding in live cells. At present, we have access to five TETA tagging tools that enable visualizing translation live (Table 1 and Figure 4). In this section, we are going to discuss how they are developed and applied for tracking translation.

**Table 1:** Summary of tandem epitope tag array (TETA) tagging tools.

Tagging tool	Type	Epitope tag	Epitope source	*K*_D_	Ref
HA-ib/HA	scFv	10xYPYDVPDYA	Influenza hemagglutinin	22 nM	[3]
FLAG-ib/FLAG	scFv	10xDYKDDDDK	*De novo* design	NA	[4]
Sun-ib/SunTag	scFv	24xEELLSKNYHLENEVARLKK	Yeast GCN4	0.38 nM	[5,6]
Moon-ib/MoonTag	Nanobody	24xKNEQELLELDKWASL	HIV gp41	35 nM	[7]
ALFA-ib/ALFA-tag	Nanobody	32xPSRLEEELRRRLTEP	*De novo* design	26 pM	[8,9]

##### HA-ib/HA and FLAG-ib/FLAG tagging tools

The HA tag and FLAG tag (Table 1) are short but strong immunoreactive epitope tags [53,56]. As the tags and their cognate antibodies were developed, they have been widely used in protein purifications and protein detections [56,57]. This is due to their superior characteristics: (1) they do not interfere with the functionality of the recombinant protein; (2) their cognate antibodies bind them with high specificity and affinity; (3) the tags can be flexibly inserted into either N-terminus, C-terminus, or in the middle of the recombinant proteins as the location does not affect the recognition by their cognate antibodies as long as they are accessible. The Looger group utilized their location flexibility and small size to develop two novel tags named smHA and smFLAG (Figure 3B), which can recruit up to 10 copies of fluorescent Fabs to a single protein molecule to significantly increase the ratio of signal-to-noise in live-cell imaging [40]. The smHA and smFLAG tags have been used by the Stasevich lab to track translation in live cells [36]. They tagged the N-terminus of their POIs with smHA or smFLAG in their reporters, then bead-loaded fluorescent Fabs in live U-2OS cells expressing the reporters to track translation (Figure 3). Later, the Stasevich group developed genetically encodable HA-ib and FLAG-ib to expand the applications of smHA and smFLAG in visualizing translation live.

The HA-ib and FLAG-ib were developed in 2019 and 2021, respectively, by Zhao et al. [3,4]. For the HA-ib development, the scFv sequence of the anti-HA antibody 12CA5 was accessed in a publication [58]. When this scFv was fused with a GFP tag and tested in live cells, it did not show any colocalization with HA-NLS (nuclear localization signal)-tagged mCherry [59]. Similarly, the original anti-FLAG-scFv-GFP did not show any enrichment in the nucleus when coexpressed with FLAG-mCherry-tagged nuclear histone protein H2B [4]. The mis-colocalization indicates the scFvs have folding defects in the intracellular environment. To improve their folding, Zhao *et al.* applied a loop grafting technique to graft all six loops from the original scFvs to other stable scFv scaffolds that have been shown to fold properly in live cells (Figure 4A1) [3,4]. Specifically, they selected five stable scFv scaffolds for loop grafting. All five scFvs have been used successfully in live cell imaging, which means their scaffolds should fold correctly in the reducing intracellular environment and their folding should not depend on the formation of disulfide bonds. After a one-step loop grafting, the same two scaffolds improved the folding of both the anti-HA and anti-FLAG scFvs. The researchers compared the scaffold sequence of the original scFvs to the selected five stable ones and found out that the original scFv scaffolds showed much higher sequence identity to the two stable scaffolds that improved folding [3,4]. The researchers mentioned that the high sequence identity could significantly reduce the risk of possible conformation changes caused by loop grafting [3,4]. This scaffold sequence identity analysis provides great guidance to quickly find the matched loops and stable scaffolds. With this guidance, the loop grafting technique becomes an efficient way to convert existing scFvs into intrabodies, as no extensive engineering and screening are needed.

The developed HA-ib and FLAG-ib are superior in live-cell imaging, especially in tracking translation in live cells. Both intrabodies show great binding specificity when labeling mature proteins in diverse intracellular environments as well as in live organisms [3,4,60,61]. The HA-ib can bind the HA tag for minutes, while the FLAG-ib shows faster turnover, which makes them suitable for different live-cell imaging applications. Both can track translation at a single mRNA level in live cells when tagging nascent polypeptide chains with the smHA or smFLAG tags (Figure 4A2) [3,4]. The diffusion of the nascent polypeptides visualized by the HA-ib/HA showed a similarity with an orthogonal tagging tool, Sun-ib/SunTag, which is described in the following section.

##### Sun-ib/SunTag tagging tool

Sun-ib/SunTag tagging tool (Table 1) is the first available genetically encodable tagging tool for tracking nascent polypeptides. To develop this tagging tool, Tanenbaum *et al*. screened three previously developed single-chain antibodies, as shown in Table 2, in live cells using a colocalization assay [5]. They attached a GFP tag to the single-chain antibodies and transfected each into cells expressing the cognate epitope-tagged mitochondrial membrane protein. They found that only the anti-GCN4 scFv/GCN4 pair showed robust colocalization at mitochondria, which indicates the binding between the scFv and the GCN4 tag. In addition, the GCN4 tag did not disrupt the organelle morphology. Therefore, the researchers chose this pair for further optimization and characterization, which is named Sun-ib/SunTag in this review.

**Table 2:** Single-chain antibodies tested for the Sun-ib/SunTag development.

Antibody	Type of probes	Epitope tag	Epitope source	Ref
C4	scFv	MATLEKLMKAFESLKSF	Human huntingtin exon 1	[62]
Vl-12.3	Single V_L_ domain (cysteine free)	MATLEKLMKAFESLKSF	Human huntingtin exon 1	[63]
Anti-GCN4	scFv	LLPKNYHLENEVARLKKLVGER (v1)	Yeast GCN4	[64]

First, the researchers tested the binding affinity of the Sun-ib to the SunTag in live cells using the Fluorescence Recovery after Photobleaching (FRAP) assay. The slow recovery in the FRAP assay indicates their tight binding, which is consistent with the low K_D_ measured *in vitro* (Table 1). Next, the researchers resolved the aggregation issue of the Sun-ib almost completely by fusing it with a superfolder GFP (sfGFP) tag [65] and a small solubility tag GB1 [66]. Then, they optimized the linker separating two SunTags to allow two Sun-ibs to bind to the neighboring tags without hindrance, resulting in a 24xSunTag array that can recruit 24 copies of Sun-ib. The ultra brightness of 24xSunTag allows imaging tagged molecules using low laser power such that the tagged molecules can be tracked for a long time. As the size of the 24xSunTag is significantly increased compared with a single GFP tag, the tagged molecules showed slower diffusion. The researchers further optimized the 22 aa SunTag (LLPKNYHLENEVARLKKLVGER, v1) to a 19 aa version (EELLSKNYHLENEVARLKK, v4). The v4 SunTag showed elevated expression in live cells due to improved α-helical propensity and reduced hydrophobicity. So far, this v4 24xSunTag (Table 1) is the most popular tool to tag nascent polypeptides and visualize translation in live cells due to its ultra-brightness (Figure 4B).

##### Moon-ib/MoonTag tagging tool

To track translation in multiple colors, the Tanenbaum lab developed another genetically encodable antibody–epitope tag pair [7] that is orthogonal to the Sun-ib/SunTag. To identify this new pair, Boersma *et al.* tested seven nanobody/epitope tag pairs that have been shown to have high binding affinity *in vitro* (Table 3). Only one pair is proven to retain its robust binding in live cells. They named this pair MoonTag system. In this review, we refer to the anti-MoonTag nanobody as Moon-ib and the epitope tag as MoonTag (Table 1 and Figure 4C).

**Table 3:** Nanobodies tested for the Moon-ib/MoonTag development.

Antibody	Type	Epitope tag	Epitope source	References
BF10	Nanobody	RTEQKDFDGRSEFAYGSFVR	Mycobacterium tuberculosis HSP16	[67,68]
CA52	Nanobody	FEDVW	Human DARC	[69]
2B2	Nanobody	EDFNMESDSFEDFWKGED	Human CXCR2	[70]
127D1	Nanobody	EDFNMESDSFEDFWKGED	Human CXCR2	[70]
54B12	Nanobody	EDFNMESDSFEDFWKGED	Human CXCR2	[70]
P2	Nanobody	HWPKPHTLWSNGVLE	Dengue virus type 2 NS1	[71]
2H10	Nanobody	KNEQELLELDKWASL	HIV gp41	[72]

The MoonTag is a 15 aa peptide (KNEQELLELDKWASL) derived from the HIV envelope protein complex subunit gp41, and the Moon-ib (clone 2H10) is a 123 aa nanobody, which is half the size of scFvs, binding to the MoonTag as tight as 35 nM (Table 1) [72]. Binding stoichiometry experiments revealed that up to ~10–12 copies of Moon-ib can bind to an array of 24xMoonTag when separated with an optimized linker. Boersma *et al.* attached the 24xMoonTag array to the N-terminus of a translation reporter (kinesin Kif18b). They visualized colocalized nascent polypeptide chains and mRNA spots lit up by the PP7/PCP system (Figure 4C). The result indicates the Moon-ib/MoonTag can work as an effective tagging tool for visualizing translation and can be coupled with other orthogonal tagging tools for tracking distinct nascent chains simultaneously in live cells.

##### ALFA-ib/ALFA-tag tagging tool

In 2019, a versatile synthetic peptide was developed via *de novo* design [8]. The researchers chose a peptide SRLEEELRRRL as it forms a stable alpha helix. To ensure net neutrality of the peptide at physiological pH, they added negative threonine and glutamic acid residues to the C-terminus. Finally, to ensure no interfering interaction between the peptide tags, prolines were added to flank the peptide sequence, resulting in the final 15 aa sequence of PSRLEEELRRRLTEP (Table 1). In homage to its structure, the tag is called the ALFA-tag [8]. To develop the nanobodies binding the ALFA-tag, the researchers immunized alpacas and selected nanobodies specifically targeting ALFA-tagged proteins by ELISA assay. The best nanobody binder was genetically modified with ectopic cysteines, allowing for a site-specific immobilization or fluorophore attachment, and is referred to as the ALFA-ib.

Initial experiments to test the nanobody as an intrabody candidate revealed efficient binding and recruitment of the ALFA-ib to different subcellular bait proteins containing the ALFA-tag [60]. Similar distribution patterns were observed when the ALFA-ib was fused to sfGFP or mEGFP, with minor occasional aggregation when fused to the latter. Using the ALFA-ib to detect ALFA-tag fused proteins in cells showed robust binding with ~10 times improved detection limit than the HA tag, the MYC tag, and the FLAG tag [8]. In line with these observations, the binding affinity of the ALFA-ib to the ALFA-tag was revealed to be ~26 pM (Table 1). The Bertrand lab and the Dufourt lab validated this strong binding in live cells later [9]. To test its application in tracking nascent polypeptide chains, the Bertrand lab and the Dufourt lab attached a 32xALFA-tag array to the N-terminus of a short mRNA transcript insulin-like peptide 4 (Ilp4) and visualized its translation (Figure 4C) [9]. The ALFA-ib/ALFA-tag has begun to prove itself as a useful tagging tool for tracking translation live.

## Visualizing translation live with single transcript resolution

With the development of TETA tagging tools, we can now visualize translation with single transcript resolution in live cells and uncover the kinetics, localization, and heterogeneity of translation (Figure 2). Although the studies used diverse tagging tools (Figures 3 and 4) in NPT, many measurements of translation kinetics show excellent agreement across several groups. For example, the five pioneer groups who developed NPT measured translation elongation rate as 3–13 aa/sec, ribosome density as 200–900 nucleotides/ribosome, and translation initiation rate as 13–50 sec/ribosome [6,35,38]. These translation parameters measured by NPT perfectly match the measurements by an orthogonal approach ribosome profiling [73,74]. This remarkable agreement across studies shows the excellent accuracy and repeatability of the NPT technology. Since then, NPT has been widely used in studying translation in live cells as well as in live organisms. In this section of the review, we will summarize what we have learned so far about translation using the NPT technology.

### mRNA structure during translation

During translation, it is almost universally accepted that the mRNA 5′ and 3′ ends are brought in proximity through a 5′ cap-eIF4E-eIF4G-poly(A) binding protein–poly(A) interaction [75]. This closed-loop model is supported by several studies and the circular mRNA structure was visualized by electron microscopy [76] and atomic force microscopy [77]. This model is further supported by Morisaki *et al.* [36], where they tracked the translation of ORFs in different lengths using NPT in live cells and observed globular, instead of elongated, polysomes. After measuring the distance of the nascent chain fluorescent signals to the 3′ UTR fluorescent signals in different-length ORFs, they found that the distance did not increase along with the increase of the ORF length. Combining their results indicates that the translating mRNAs form a circular structure instead of a stretch-out structure.

However, this closed-loop model was questioned by another two studies, in which they found translating mRNAs were more open and stretched out compared with nontranslating mRNAs applying single-molecule fluorescence in situ hybridization in fixed cells [78,79]. These results were further validated by a live-cell imaging study, in which Koch *et al.* applied NPT to study cap-initiated and IRES-initiated translation in a single mRNA and found that loading of ribosomes caused mRNAs to stretch out [50]. These discrepancies demonstrate that the classic closed-loop model needs to be revised and reexamined.

### Subcellular local translation

Targeting mRNAs to diverse subcellular locations is a mechanism that cells utilize to spatiotemporally regulate gene expression. Localization to certain subcellular sites can alter translation efficiency. The Chao lab applied the Sun-ib/SunTag tagging tool to visualize the translation of a cytosolic protein *Renilla* luciferase and revealed a population of ER-associated mRNAs with an increased number of translating ribosomes compared with the same mRNAs translating in the cytosol [30], indicating that more efficient translation of cytosolic proteins can occur at the ER.

The induction of ER stress via thapsigargin to repress protein synthesis throughout the cell leads to the protection of active translation localized at the mitochondria [80]. In this study, the researchers visualized the translation of various subcellular locations targeting reporter mRNAs with NPT. When under ER stress, the active translation was reduced by 73% at the ER and 50% in the cytoplasm, but only by 27% at the mitochondria. The researchers demonstrated that the mitochondrial protein ATAD3A interacts with PERK and competes with the translation initiation factor eIF2α. Furthermore, bulk proteomic analysis revealed that ATAD3A rescues a subset of mitochondrial proteins during ER stress, indicating increased PERK–ATAD3A interactions during ER stress that facilitate mitochondria–ER contact sites may be protective and allow for continued translation even during cellular stress.

Application of NPT in neurons has revealed that actively translating mRNAs can be transported in neuron dendrites [3,37]. Wu et al. showed the percentage of translating mRNAs decreased from ~40% in proximal dendrites (<30 µm from the soma) to ~10% in distal dendrites (>100 µm from the soma), indicating translation repression in distal dendrites [35]. Investigation of mRNAs that target to extending protrusions of migrating mesenchymal cells revealed that though these mRNAs undergo translation at the protrusions, they are suppressed and clustered in retracting protrusions [81]. These studies provide solid evidence that translation in polarized cells like neurons is spatially regulated.

### Translation in membraneless organelles

Membraneless organelles containing mRNA clusters, such as SGs and P-bodies, are known to play a critical role in regulating gene expression. SGs are formed under cellular stress, the formation of which coincidences with global silencing of translation. Therefore, it is often speculated that SGs are contributing to the translation repression under stress. However, little direct evidence supports this model until the study by Moon et al. [51]. Using NPT, they revealed the translation of a KDM5B reporter declined following stress, and 98% of SG-associated mRNAs were translationally repressed, while 1% and 2% of SG-associated mRNAs still retained nascent chains. After imaging with higher temporal resolution, they observed that the translating mRNAs interacted with SGs transiently, while nontranslating mRNAs formed stable interactions within the granules. After removing the cellular stress, translation resumed within minutes of the SGs dissipating. However, the model is questioned by another single-molecule study. The Chao lab has shown that translation can occur in SGs utilizing the Sun-ib/SunTag NPT technology [82]. They placed the 5′ UTR of ATF4 in front of the 24xSunTag array, which allowed the mRNAs to be efficiently translated during cell stress while other mRNAs were translationally repressed. Surprisingly, the mRNAs localized to SGs underwent complete translation cycles, indicating that the SG microenvironment contains the machinery for each step of the translation cycle, and mRNA localization to SGs does not require inhibition of translation. Moreover, they revealed that translating mRNAs could remain localized in SGs for more than 5 min, which is the opposite of the transit interaction observed by Moon et al. [51]. As ATF4 is up-regulated during cell stress, to determine if the SG-associated translation also happens in repressed transcripts during stress, they swapped the ATF4 5′ UTR with the 5′ TOP motif of a ribosomal protein L32 which is translation inhibited during cell stress. They observed a significant decline in the number of translating mRNAs, from 76% to 2%, after inducing cell stress. However, few translating mRNAs existed in both cytosol and SGs during cell stress. There was no location preference for translating mRNAs during stress. As the two NPT studies show debatable results, the role of SGs in regulating translation is still unclear to us and needs further investigation.

In another study, the Stasevich lab tethered the Argonaute2 protein to the 3′ UTR of the NPT reporter to silence translation, reflecting the endogenous microRNA-mediated gene silencing [52]. They observed translationally silenced mRNAs clustered and coalesced with P-bodies where the mRNAs maintained a translationally silenced state [52]. However, after translation silencing, mRNA decay was not observed for mRNAs located either in or out of P-bodies [52]. Similarly, the Singer lab recruited Argonaute2 through a microRNA miR-21 to the 3′ UTR of their NPT reporter and observed translation silencing prior to mRNA decay in fixed cells [83]. However, they did not study if the translation-silenced mRNAs were clustered and decayed in P-bodies. Further investigations on how P-bodies regulate gene expression are needed.

### Translation bursts

Translation bursts have been implied by several NPT studies [35,36]. However, little is known about the kinetics and regulation due to the challenge of tracking highly mobile cytoplasmic mRNA for multiple translation events. The Wu lab tethered the NPT reporter mRNAs on the plasma membrane through the interaction of 12xPP7 stem-loops embedded in the 3′ UTR of the reporter mRNAs and plasma membrane-localized PCP, besides using the Sun-ib/SunTag for tracking nascent chains and the MCP/MS2 for tracking single mRNAs [84]. This tethering strategy allowed them to track single mRNAs for multiple complete translation events with a minimal fluorescence background. From these long tracks, they observed that mRNAs switched between active (burst) and inactive (dwell) states, indicating that translation occurred in a bursting manner instead of constitutively. They measured the mean durations of burst and dwell are 43.3 ± 10.3 and 3.5 ± 1.5 min, respectively. Then, they demonstrated that bursts could overlap as the measured burst lasting time was increased in longer ORF reporters, suggesting the measured burst time might be longer than the intrinsic translation active state of mRNAs. Furthermore, they found that the structure of 5′ UTR modulated burst amplitude without any effect on burst frequency; in contrast, the 5′ TOP RNA sequence was shown to alter the temporal dynamics, instead of the amplitude, of the burst. Inhibition of mTOR signaling using Torin-1 significantly increased the dwell time without perturbing the intrinsic burst time. This study demonstrates that translation occurs in bursts instead of constitutively and is the first to uncover the kinetics and regulation of translation bursts.

### Noncanonical translation

In eukaryotic canonical translation, mRNAs initiate translation through a cap-dependent mechanism that requires the binding of 5′ cap by the eukaryotic initiation factor 4F complex, the AUG start codon recognition by the recruited 43S ribosomal subunit, and the following recruitment of 60S ribosomal subunit to make an 80S elongation-competent ribosome [1]. However, under certain conditions, translation can occur in a noncanonical manner. Recently, the NPT technology has been applied to investigate the kinetics and the heterogeneity of noncanonical translation, such as frameshifting, IRES-initiated translation, alternative start codon-initiated translation, and repeat-associated non-AUG (RAN) translation.

Frameshifting is caused by slippery sequences, which lead to translation slips by ±1 nucleotide, resulting in two distinct nascent chains from a single mRNA. Viruses exploit this frameshifting mechanism to minimize their genomes and efficiently synthesize the proteins needed for replication. The Stasevich lab developed a frameshifting reporter that encoded 12xFLAG tagged AlexX in the 0 frame and 12xSunTag fused to XXLb1 in the −1 frame. They applied this reporter to study the frameshifting dynamics of an HIV-1 frameshift sequence [49]. They revealed that a subset of ribosomes (~8%) robustly frameshifted and frameshifted translation occurred in bursts at similar elongation rates (~3 aa/sec) to in-frame translation [49]. Furthermore, frameshifted ribosomes displayed longer pauses at the frameshift sequence that led to ribosome traffic jams, likely resulting in the continuation of the frameshifted translation.

IRES sequences can recruit ribosomes and initiate translation in a cap-independent manner [85]. Viruses often use IRES to hijack host ribosomes and promote cap-independent translation. The Stasevich lab applied a bicistronic sensor to study the encephalomyocarditis IRES-initiated translation and cap-initiated translation simultaneously in live cells [50]. The bicistronic biosensor consists of an mRNA transcript encoding for a smFLAG tagged KDM5B for 5′ cap-initiated translation tracking and a 24xSunTag tagged Kif18b following the IRES sequence for IRES-initiated translation tracking [50]. They found that the bursts of IRES-initiated translation were shorter and rarer than the cap-initiated translation, which was caused by fewer ribosomes recruited by IRES instead of an altered translation elongation rate. However, under stress conditions, IRES-initiated translation was unaffected or even up-regulated, while the cap-initiated translation was drastically down-regulated [50].

In addition, two other studies have investigated how viral mRNAs translate in host cells applying NPT. One study focuses on studying full-length HIV-1 mRNAs [86] and the other focuses on +RNA viruses [87]. The findings from these studies help us understand the underlying mechanisms by which viruses hijack host cell ribosomes during infection.

Typically, translation initiates at the most upstream AUG start codon. However, studies have shown that translation can be initiated at alternative start codons. Many mRNAs contain multiple start codons, including upstream open reading frames (uORFs). Selection of correct translation start sites is critical for synthesizing correct protein products. The Tanenbaum lab applied two orthogonal nascent chain tagging tools, Sun-ib/SunTag and Moon-ib/MoonTag, to study reading frame selection [7]. The reporter in this system contains 36xMoonTag and 36xSunTag fused in an alternating fashion and positioned in different ORFs (the MoonTags are in the 0 reading frame and the SunTags are in the −1 reading frame) with their distinct start codons. This MoonTag and SunTag hybrid tag is named MashTag. MashTag enables imaging of two distinct nascent chains tagged either by the MoonTag or the SunTag initiated at two different start sites. They observed most mRNAs were strongly translated in the MoonTag frame, 66% of mRNAs showed both SunTag and MoonTag translation events, and widespread variability (0%–100%) in the frequency of the out-of-frame (OOF) translation. They demonstrated that OOF translation of the SunTag reporter is predominately caused by alternative start site selection near the 5′ end of the ORF [7]. They further applied the MashTag to study how uORF regulated the main ORF. Their results show that most mRNAs go through both leaky scanning and translation reinitiation mechanisms, but a small fraction of mRNAs prefer one mechanism over the other [7].

Translation can be initiated through a noncanonical RAN initiation mechanism. The Wu lab applied NPT to study the RAN translation of an expanded RNA sequence GGGGCC in the *C9ORF72* gene [88], the abnormal expansion of which can be translated into five different toxic dipeptide-repeats: GA, GP, GR, PR, and PA that causes amyotrophic lateral sclerosis and frontotemporal dementia. To investigate the biogenesis mechanisms of these dipeptide-repeats, the Wu lab inserted 70xGGGGCC repeats with the 113 nt upstream intron sequence in front of 24xSunTag. The SunTag was placed in the same reading frame as the GA, GP, or GR. They observed that the RAN translation occurred in each frame but was significantly less efficient than canonical translation. The RAN translation happened more efficiently in the GA frame than in the GP or GR frame, which was shown to be caused by the extra initiation of the GA frame through a near-cognate CUG start codon located 24 nt upstream of GGGGCC repeats. Then, the Wu lab generated a bicistronic reporter to study if frameshifting could occur in the expanded sequence. They flanked a 24xHA tag-GGGGCC repeats with an AUG start codon and a stop codon, followed by a 24xSunTag lacking the AUG start codon in either the +1 or + 2 reading frame, then followed by an in-frame stop codon. The 24xHA tag would be translated canonically (frame 0), if frameshifting occurred in the GGGGCC repeats, the 24xSunTag would be translated (frame +1 or +2). Their results showed that 90% of the time, mRNAs were translated in their original frame without frameshifting. When frameshifting did happen, it occurred more often for GR to GA (6.4%), then GA to GP (4.3%), and finally, GA to GR (2.3%). The ribosome run-off experiment showed that the translation elongation rate in the GP and GR frames was 2.3- and 2.8-fold slower than in the GA frame. The slow elongation rate in the GR frame could lead to more ribosome collision and subsequent more frequent frameshifting. Furthermore, they found that the encoding amino acids sequence, rather than the secondary structure of the GGGGCC repeats, resulted in the difference in the translation elongation rate of the frames. Knocking down the ribosome-associated quality control factors ZNF598 or Pelota further slowed down the elongation rate of both GGGGCC repeats encoded GR and GA, but did not show an effect on randomized codon encoded same dipeptide-repeats. The results indicate that the repeated RNA sequence or structure, not the dipeptide-repeats sequence, invokes the quality control mechanism.

In summary, while noncanonical translation events help increase cellular proteome diversity [89,90], these events can also occur with translational errors [90,91] and potentially result in incorrect and/or dysfunctional polypeptides. Therefore, understanding the regulation mechanisms of noncanonical translation is critical for improving translation fidelity. The NPT technology provides a powerful method to directly and accurately measure the kinetics of noncanonical translation with single mRNA precision, which enables us to uncover the regulation mechanism of noncanonical translation.

### Ribosome-associated quality control

Translation of a problematic mRNA sequence can induce ribosome stalling which triggers ribosome-associated quality control (RQC) including ribosome rescue and nascent polypeptide degradation. The Wu lab and the Green lab applied the Sun-ib/SunTag NPT technology to study the kinetics of RQC triggered by ribosome stalling of poly(A) sequence with single mRNA resolution [92]. Their results show that the poly(A) sequence causes ribosome stalling and results in long queues of collided ribosomes. The ribosome loading of the poly(A)36 and poly(A)60 reporters is 3- or 4-fold higher than that of the no poly(A) control. The total protein output of the poly(A)36 or poly(A)60 is reduced 6- or 9-fold, which is mainly contributed by RQC. Interestingly, the poly(A) reporters show a similar percentage of actively translating mRNAs compared with the no poly(A) control and can undergo many rounds of translation and ribosome rescue. They used translation initiation inhibitor harringtonine to synchronize the run-off time of active translating ribosomes and were able to determine that the ribosome rescue time of the poly(A)36 reporter is ~5 min and the poly(A)60 reporter is ~8 min. Then, they repressed the translation initiation by inserting an ATF4 5′ UTR to their reporters and found out that the repression of translation initiation resulted in significantly decreased RQC efficiency by shortening the queue length of collided ribosomes. Furthermore, they uncovered that ZNF598 accelerated the clearance of stalled ribosomes. This study is the first to unveil the kinetics of RQC and the role of ZNF598 in regulating the kinetics of ribosome rescue, indicating the powerful NPT technology can further elucidate our understanding of RQC.

### Nonsense-mediated decay

One way to regulate gene expression is through surveillance of transcript integrity. The nonsense-mediated decay (NMD) surveillance system degrades transcripts containing premature termination codon (PTC), which prevents the synthesis of truncated and potentially toxic proteins. Visualization of individual mRNAs undergoing NMD in real time was accomplished in 2019 by the Tanenbaum lab [93]. They used the Sun-ib/SunTag translation tracking technology to track when, where, and how often NMD could happen. Their results showed that while NMD efficiency was affected by the number and position of introns around the PTC as well as the exon sequence downstream of the PTC, each round of translation had an equal probability of inducing NMD of an mRNA molecule. They also showed that nuclear cap-binding complex (CBC)-bound mRNAs and cytoplasmic cap-binding protein eIF4E-bound mRNAs underwent NMD at similar levels, which for the first time provided evidence that NMD does not occur preferentially on CBC-bound mRNAs. Interestingly, a subset of mRNA NMD reporter molecules (5%–30%) were resistant to NMD-dependent cleavage, indicative of heterogeneity in NMD regulation mechanisms. Furthermore, they revealed the decay kinetics of the 3′ fragment after mRNA cleavage by XRN1 as 38 nt/sec. Moreover, after precise measurement of decay kinetics using a long 3′ UTR reporter, two populations decaying with different rates were observed. They hypothesized that the fast-degrading mRNAs might need a single XRN1 binding event, while the slow-degrading mRNAs might require two or more XRN1 binding events for complete RNA degradation. This hypothesis was supported by showing that the decay rate of the fast-degrading mRNAs was not significantly affected by XRN1 depletion, while the slow-degrading mRNAs decayed substantially more slowly. This study demonstrates that the NPT technology provides a powerful method for studying NMD by revealing key determinants of NMD variability and efficiency.

### Translation of endogenous genes in live cells and live organisms

The NPT technology, coupled with genome editing techniques, has been used to track the translation of endogenous genes. The Berthoud lab attached a 56xSunTag to the endogenous housekeeping gene POLR2A via CRISPR/Cas9 allowing for the first observed translation of endogenous transcripts and quantification of translation elongation rate as 13–18 aa/sec and ribosome density as 1.3/kilobase [38]. Later tracking the translation of the tagged heavy chain of dynein 1 (DYNC1H1) revealed the existence of translation factories and showed that DYNC1H1 polysomes were actively transported by motors. Interestingly, when researchers repeated the tagging of the endogenous POLR2A with a 32xSunTag or 32xALFA-tag, they observed ~2.5 times brighter signals from the ALFA-tag system [9], indicating that the ALFA-tag might be an integral tool to continue the illumination of endogenous translation events.

Furthermore, the Sun-ib/SunTag system has also been integrated into *Drosophila* embryos revealing translation during embryo development. The Ashe group applied the Sun-ib/SunTag system to study the translation of zygotically transcribed Hunchback mRNAs, which is crucial for anterior–posterior patterning [94]. They found out the translation started from uniform in the embryos, then declined from the anterior until it was restricted to a posterior band in the expression domain. Dufourt and Lagha studied the translation of the conserved major epithelial–mesenchymal transition-inducing transcription factor Twist [95]. They identified spatial heterogeneity in translation efficiency and revealed translation factories at basal perinuclear regions, where clustered mRNAs are preferentially co-translated. The Lehmann lab uncovered that germ granules, specialized ribonucleoprotein (RNP) granules in early *Drosophila* embryos, are the active translation site of a maternally deposited *nanos* mRNA, although these mRNAs are present in the entire embryo [96]. Their results demonstrate that RNP granules not only serve as a storage hub for translationally repressed mRNAs but also activate translation.

More recently, the Sun-ib/SunTag and the ALFA-ib/ALFA-tag tagging systems have been further optimized and applied for tracking translation in *Drosophila* embryos [9]. The Bertrand lab and the Dufourt lab tagged a repeated SunTag array to the N-terminus of the endogenous Twist gene and a repeated ALFA-tag array to the N-terminus of another endogenous gene insulin-like peptide 4 (Ilp4) in *Drosophila* embryos. They succeeded in imaging the translation of the two genes in live embryos simultaneously. In addition, they were able to clearly distinguish the localization of Ilp4 protein in the cytoplasm from Twist protein in the nucleus of live embryos, indicating that the Sun-ib/SunTag system and the ALFA-ib/ALFA-tag system are orthogonal to each other and therefore are suitable for simultaneously imaging translation in a multiplex manner. As these tags continue to be optimized for live model organisms, we expect an influx of endogenous translation experiments to continue to explore gene regulation nuances between different model organisms as well as between different cell types of those model organisms.

## Concluding remarks

The ability to study the kinetics of translation non-invasively and in the native intracellular environment has begun to offer new insights and novel discoveries of translation regulatory processes. Here, we have shown the great strides the nascent polypeptide tagging technology has made since its development in 2016. The NPT technology uses fluorescent intrabodies and their cognate tandem epitope tags to track nascent chains directly and has illuminated translation kinetics, locations, heterogeneity, and regulation in live cells. The combination of the TETA tagging tools furthers the study of noncanonical translation, including frameshifting, IRES-initiated translation, ORF selection, and RAN translation, which have provided new insights into how proteomic diversity is achieved at the translation level. Recently, researchers have even begun to assess the translation kinetics, heterogeneity, and spatial regulation in model organisms. The continued development of intrabodies that recognize specific forms of a protein, like correctly folded forms of a protein and specific modifications of a protein, will enable us to visualize the whole lifecycle of a protein and, therefore, will continue to refine our understanding of protein regulation in the native live cellular environment.

## Abbreviations

ELISA: enzyme-linked immunosorbent assay
ER: endoplasmic reticulum
FP: fluorescent protein
FRAP: fluorescence recovery after photobleaching
FUNCAT: fluorescent noncanonical amino acid tagging
Fab: antigen-binding fragment
FabBL: Fabs-bead loading
Fc: crystallizable fragment
GFP: green fluorescent protein
IRES: internal ribosome entry site
MCP: MS2 coat protein
NMD: nonsense-mediated decay
NPT: nascent polypeptide tracking
OOF: out-of-frame
OPP: O-propargyl-puromycin
ORF: open reading frame
PCP: PP7 coat protein
POI: protein of interest
PTC: premature termination codon
P-body: processing body
RAN: repeat-associated non-AUG
RFP: red fluorescent protein
RNP: ribonucleoprotein
RQC: ribosome quality control
SG: stress granule
SRP: signal recognition particle
TETA: tandem epitope tag array
THRONCAT: threonine-derived noncanonical amino acid tagging
TOP: terminal oligopyrimidine
TRICK: translating RNA imaging by coat protein knockoff
UTR: untranslated region
UV: ultraviolet
aa: amino acid
ib: intrabody
nb: nanobody
ncAA: noncanonical amino acid
scFv: single-chain antibody fragment
uORF: upstream open reading frame
